# Microstructural and Thermo-Physical Characterization of a Water Hyacinth Petiole for Thermal Insulation Particle Board Manufacture

**DOI:** 10.3390/ma12040560

**Published:** 2019-02-13

**Authors:** Adela Salas-Ruiz, María del Mar Barbero-Barrera, Trinidad Ruiz-Téllez

**Affiliations:** 1 Department of Construction and Technologies in Architecture, Universidad Politécnica de Madrid, Avd: Juan Herrera 4, 28040 Madrid, Spain; mar.barbero@upm.es; 2 Botany Area, Faculty of Science, Extremadura University, Avd Portugal 0, 06006 Badajoz, Spain; truiz@unex.es

**Keywords:** Water Hyacinth, cellulose fibres, bio-based thermal insulation, binder-less, anatomy characterization, chemical characterization, thermal conductivity, mechanical characterization, invasive Plant

## Abstract

Water Hyacinth (*Eichhornia crassipes*) is a dangerous and invasive aquatic species, of which global concern has sharply risen due to its rapid growth. Despite ample research on its possible applications in the construction field, there are no clear references on the optimal use of the plant in finding the most efficient-use building material. In this paper, a microstructural and chemical characterization of the Water Hyacinth petiole was performed, in order to find the most efficient use as a construction material. Subsequently, two types of binder-less insulation panels were developed, with two types of particle size (pulp and staple). A physical, mechanical, and thermal characterization of the boards was performed. These results demonstrated that it is possible to manufacture self-supporting Water Hyacinth petiole panels without an artificial polymer matrix for thermal insulation. The boards showed good thermal conductivity values, ranging from 0.047–0.065 W/mK. In addition, clear differences were found in the properties of the boards, depending on the type of Water Hyacinth petiole particle size, due to the differences in the microstructure.

## 1. Introduction

A growing and global concern about energy consumption has sparked the search for new alternatives for artificial materials. In the construction sector, the use of bio-based materials is one of the key points in reducing the energy consumption associated with fabrication [1], while improving energy efficiency during the time of use of the building. Interest in biomass-based insulation materials has increased in recent years, and this is reflected in the number of relevant publications [2]. As bio-based construction materials for thermal insulation, natural fibres have the advantage (over inorganic or petroleum-based ones) of low environmental impact. For example, some specific environmental benefits are their non-toxic nature and biodegradability [3].

The physical and mechanical properties of plant-based fibres are different, depending on their botanical origin. Each species and tissue has its corresponding anatomy. Cross-sections may oscillate between 0.3–0.4 mm^2^ [4]. They are formed by walled plant cells, organized into tissues. The tissues forming the fibre are mainly vascular bundles (xylem and phloem), accompanied by supporting tissue and some ground tissue. The former has many empty cells, only formed with walls, and constitute microscopic channels or tubes inside the plant, which remain present in the interior of the dried raw material [5].

Plant fibres are composites, microstructurally comprised mainly of hollow cellulose fibrils entrenched together by lignin and the hemicellulose matrix [6]. The walls of the cells, which physically constitute the fibre, have a biochemical composition that is quite diverse with respect to their species and origin. In some cases, the cellulose content is higher than 50% (even up to 60%–70%); in others, the lignin content reaches about 40%. Hemicellulose values of about 20% are frequent [6,7]. Cellulose determines the tensile strength of the material; lignin is responsible for resistance against biodegradation; and hemicellulose binds the cellulose polymers, provides secondary strength, and absorbs moisture [8]. Other physical parameters of these chemical biopolymers also condition the fibre properties, but the mechanical and thermal characteristics of a plant fibre are mostly determined by its microscopic anatomy, the distribution of the cells forming the fibre tissue, and the type of cells that constitute it. Plant histology ends up affecting such fibre properties as thickness, density, porosity, rigidity, resistance, conductivity, and air permeability [9]. These attributes make each natural fibre more suited to one industrial purpose or another. 

Bibliographic reviews of plant fibres, as employed for the manufacture of thermal insulation construction elements [1,2,10,11,12,13], include nearly twenty types (bagasse, bamboo, banana, barley, cattail, coir, corn, cotton, date, durian, elephant grass, flax, hemp, jute, kenaf, palm, pineapple, reeds, rice, sanseviera, sisal, straw, and sunflower). They are obtained from steams, leaves, culms, or other parts of a varied source of plant species [6]. Some of these are typically grown extensively for profit (banana, barley, coir, corn, date, palm, pineapple, rice), while others are non-crop plants (bagasse, bamboo, cotton, durian, elephant grass, hemp, jute, kenaf, reeds, sanseviera, sisal, straw, sunflower).

In contrast, the use of invasive species allows their eradication, with some benefits to the community in which it is generated. This is the case of Cattail Narrow [14,15,16,17] or Water Hyacinth (WH) [18].

WH (*Eichhornia crassipes*) is a dangerous and invasive aquatic species, which has garnered global attention due to its rapid growth. It is a plague in most of the tropical countries of the world. Its high water content (about 90%–95%) made management difficult and unprofitable, in such a way that millions of dollars have been spent every year on removing tons of biomass of this plant from bodies of water [19]. Although it was used in the handicraft sector [20], Water Hyacinth Petiole (WHP) as a construction material has been studied since the 1980s, when plant fibres appeared as an alternative solution to asbestos [21,22]. In addition, it has been analysed as a reinforcement material in concrete, mortars, and cement pastes [23,24,25,26,27], geotextiles [28], soil [8,29,30], or plastic matrices [31,32]. However, its inefficient use in the sector is due to the lack of a complete understanding of the anatomy, structure, and chemical composition of WHP. 

There are no clear references about the relationship between a whole characterization of WH and its efficient use as a building material. In terms of thermal insulation properties, previous studies used WH to make insulation panels, blended with natural rubber [33] or cement [34,35], and they obtained good thermal properties. However, a WH characterization in depth is necessary, in order to find an efficient use of WH in the construction sector, such as in insulation material.

Based on the above, the main goal of this paper is to study the correlation between the microstructure and chemical characterization of the WH, in order to find its optimal use as a thermal insulation material. Based on chemical results, a WH panel (without additives) was analysed in terms of its physical, mechanical, and thermal behaviour. 

## 2. Materials and Methods

WH Petioles (WHP) were used for this research. The plants were collected in the Guadiana River, Badajoz, Spain, in November 2015 and identified (as *Eichhornia crassipes*) in the Botany Area of the University of Extremadura (Badajoz). Permission for Scientific Research was obtained (General Directorate of Environment Reference Number CN35/15/ACA). Vouchers are conserved at the UNEX (Universidad de Extremadura) Herbarium.

The petioles were separated from the roots and other parts of the plant, and washed thoroughly in order to remove undesirable materials on the surface. Dried WHP was used for all the tests, except for the internal structure observation. The WHP were sun-dried in the Botany Department for two weeks at 30 °C, to achieve up to a 95% loss in weight.

### 2.1. WH Petiole Characterization

#### 2.1.1. Microstructural Observations

The internal structure of the WH material was observed at UNEX, using a scanning electron microscope (SEM) HITACHI S4800, equipped with a BRUKER XFlash Detector 5010 energy dispersive X-ray (EDX), at a voltage of 15 kV. Transversal sections at the medium of the total length of the fresh WHP were made using a sharp razor blade, with a fresh blade. The WHP were fixed in the sample carrier by graphite double-sided adhesive tape and then metallized with gold. Measurement values were determined by analysing at least 50 different areas of each sample.

#### 2.1.2. Moisture Content

Physical WHP moisture content parameters were measured, since they clearly influence the final material [12,36]. The methodology followed for their determination is described below. An electronic balance with a precision of 0.001 g was used and the test was repeated 50 times for both WHP size groups, in order to reduce dispersion of the results.

After the drying process (described above), the measured weight (M_n_) was registered. Afterwards, the WHP was put into an oven at 60 °C, and weight measurements were carried out over 24 h. Dry weight (M_d_) was considered achieved when three subsequent measurements had a deviation lower than 1%. Average moisture content was calculated using the equation:

(1) $$\mathrm{Moisture}\;\mathrm{content}\;\left(\%\right)=\frac{M_{n}-M_{d}}{M_{n}}\times 100\%.$$

#### 2.1.3. Water Absorption

The water absorption coefficient was calculated in order to determinate the hygroscopicity capacities of the WHP. An electronic balance with a precision of 0.001 g was used and the test was repeated 50 times for both WHP size groups. The WHP was submersed in a water tank at 22 °C. After 24 h, the WHP was removed from the water, and the saturated weight registered (M_sat_). 

The average water absorption was calculated as the percentage of weight increase, following the equation:

(2) $$\mathrm{Water}\;\mathrm{absorption}\;\left(\%\right)=\frac{M_{\mathrm{sat}}-M_{d}}{M_{d}}\times 100\%.$$

#### 2.1.4. Bulk Density

The WHP samples were picked up randomly and dried in an oven at 60 °C. Dried weight (M_d_) was considered when three subsequent measurements at 24 h had a deviation lower than 1%. Afterwards, the WHP was submersed in a water tank at 22 °C. The initial volume introduced (V_w0_) was measured. After 24 h, the WHP was removed from the water and the saturated weight registered (M_sat_) as well as the final water volume (V_wf_).

Bulk density was determined using the equation:

(3) $$\mathrm{Density}\;\left(g/{\mathrm{cm}}^{3}\right)=\frac{M_{d}}{V_{wf}-V_{w0}+\frac{M_{\mathrm{sat}}-M_{d}}{\rho w}},$$

where _w_ is the density of water at 22 °C.

#### 2.1.5. Chemical Characterization

The WHP was crushed and sieved through a #40 mesh (0.40 mm) standard sieve, in accordance with TAPPI T 257 [37]. Then, the moisture content of the samples was calculated, as explained above. Four samples and two blank tests were prepared in each test. 

Chemical characteristics, regarding the waxes, were evaluated by the standards TAPPI T 204 om-88 [38]. Dried samples were placed into a tared extraction thimble and both were introduced into a Soxhlet extraction apparatus which was then connected to an extraction flask with natural organic solvent. The extraction apparatus was connected, and the extraction flask placed into a water bath set at 70 °C for 4 h. Afterwards, the flasks were removed and the extract was transferred to a tared dish. The dish and the extract were dried in an oven at 105 °C. The extracted content was calculated as the percentage of extract weight to the initial dry weight of the WHP.

Hot water-soluble materials were evaluated by the standards TAPPI 207 om-99 [39]. Samples must be free of organic solvent material, and therefore the WHP residues of the previous test were utilized. They were placed in Erlenmeyer flasks with hot distilled water. The flask was connected to a reflux condenser for 3 h, making sure that the water level of the bath was held above the stock level in the flask. Afterwards, the contents of the flask was transferred to a tared filtering crucible. Samples were washed with hot water and then introduced into an oven at 105 °C for 24 h. Hot water-soluble material was determined by comparing the initial weight mass of the test specimen and the weight of the samples after extraction.

Holocelullose was determined by Wise’s Methods [40]. Samples must be free of organic soluble materials as well as hot water-soluble materials. Therefore, the residues of the previous test were utilized. Samples were introduced into a boiling flask with distilled water, 1.5 g of NaClO_2_, and 10 drops of CH_3_COOH. The flasks were connected to a distillation apparatus and were placed in a boiling water bath at 80 °C for 4 h. During this time, 1.5 g of NaClO_2_ and 10 drops of CH_3_COOH were added each hour. Afterwards, the boiling flasks were introduced into an ice water tank, until the samples were cool. The contents of the flask was transferred to a tared filtering crucible. The solid residue was washed with distilled cool water and then introduced into an oven at 105 °C for 24 h. Holocellulose was determined by comparing the initial weight mass of the test specimen and the weight of the samples after extraction.

Finally, carbohydrates and lignin were determined, according to the NREL Laboratory Analytical Procedure [41]. As in the case of the holocellulose determination test, this procedure is suitable for samples that do not contain extractive material. Samples were placed into a tared pressure tube with 72% HCl. Pressure tubes were placed in a water bath set at 30 °C for 60 min. The samples were stirred every 10 min, without removing them from the bath. Upon completion of hydrolysis, the acid was diluted to a 4% concentration by adding deionized water. The tubes were placed in an autoclave for 1 h at 121 °C. After completion, the solution was transferred into a tared filtering crucible and was vacuum filtered. The filtrate was captured in the filtering flask. The insoluble residues were washed with deionized hot water and dried at 105 °C, until a constant weight was achieved. The lignin content was determined by comparing the initial weight mass of the test specimen and the weight of the samples after extraction. Monosaccharides were determined by High Performance Liquid Chromatography (HPLC) using a BECKMAN System Gold 126 HPLC chromatograph provided with a BIO RAD Amiex HPX-87 P column, which was set to 85 °C in a column oven.

### 2.2. WHP Board Characterization

**Figure 1:**
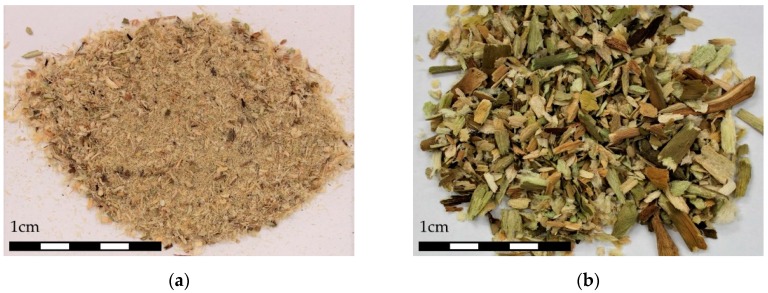
Final Water Hyacinth Petiole (WHP) appearance after the crushing process: pulp (**a**) and staple (**b**).

Based on the WH chemical and anatomic characterization, two types of boards (without additives) were constructed, with different particle sizes. Dried WHP were manually crushed and then sieved through a #2mm standard sieve (R20). Subsequently, they were categorized into 2 groups: pulp WHP (Figure 1a) (WHP passing through #2 sieve, R20) and staple WHP (Figure 1b) (WHP retained on #2mm sieve, R20). “Pulp” and “staple” designation were determined according to [42].

#### 2.2.1. Particle Board Manufacture

**Figure 2:**
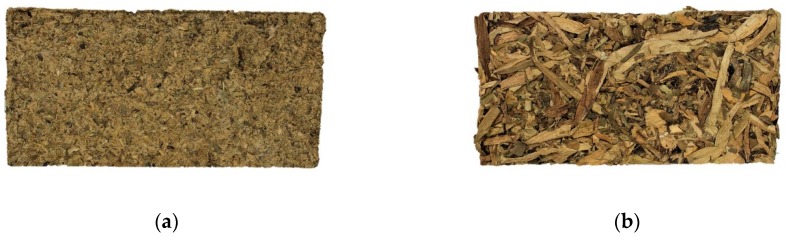
Manufactured Binder-Less WHP panels: (**a**) pulp WHP: Panel made with pulp (WHP passing through #2 sieve, R20); (**b**) staple WHP: Panel made with staple (WHP retained on #2 sieve, R20).

WHP board was blended without any artificial adhesive, taking advantage of the naturally occurring polysaccharides in the plant. The WHP was mixed with water for 300 s, for homogenization. According to previous empirical tests, developed by the authors, the water to WHP ratio was fixed at 1.75, in order to ensure proper WHP particle wetting. The mix was then poured into a frame of dimensions 150 × 150 × 15 mm, wrapped in a plastic low-density polyethylene layer. The board was compressed at 40 Pa for 24 h, and then were kept at 22 ±1 °C with 50% ±10% relative humidity. The final appearance is shown in Figure 2. Tests were performed at 28 days or curing time.

#### 2.2.2. Microstructural Observations

Board microstructure was observed by SEM-EDX, using the same methodology as in the petiole characterization. Samples were cut using a sharp razor blade, with a fresh blade. Afterwards, they were fixed in the sample carrier with a bi-adhesive graphite sheet, and then metallized with gold.

#### 2.2.3. Bulk Density of WHP Boards

Bulk density is useful in predicting thermal insulation behaviour [11,12]. It was calculated as the coefficient between the mass and volume of the sample. It was measured at 28 days under laboratory conditions (22 ±1 °C and 50% ±10% relative humidity). Both an electronic balance with a precision of 0.001 g and a digital micrometre VOGEL DIN 862 with a resolution of 0.01 mm were used. The European standard 1602 [43] was followed, and the result was given as an average of 12 measurements. 

#### 2.2.4. Moisture Content of WHP Boards

Moisture content (MC) was calculated following the European standard 322 [44]. WHP board weight was registered under laboratory conditions (22 ±1 °C and 50% ±10% relative humidity). Afterwards, they were introduced into an oven at 103 ±2 °C, and weight measurements were carried out over 24 h. Dry weight was achieved when three subsequent measurements had a deviation lower than 1%. An electronic balance with a precision of 0.001 g was utilized. MC was calculated using Equation (3), and the result was given as an average of 12 measurements. 

#### 2.2.5. Water Absorption of WHP Boards

According to the European Standard 12087 [45], water absorption by total immersion (WA) was calculated. It was based on the measurement of the saturated samples compared to the dried weight. A total of 10 samples per WHP board type were submersed in a water tank at 22 °C. Due to the high absorptivity of the panels, the test was finished after 24 h, at which point the water was removed and filtered. The saturated weights of the samples were collected, and then they were introduced into an oven at 103 ±2 °C. When the masses of the samples were constant, the dry weight was registered. The result was expressed as an average of percentage of increase, following Equation (2). In both cases, the loss of material when they were submerged into water meant that the test had to be performed with a geotextile. 

#### 2.2.6. Thickness Swelling of WHP Boards

Swelling after immersion in water was measured according to the European Standard 317 [46]. Thickness Swelling (TS) was calculated as a percentage of increase between before and after submerging the samples in water for 24 h. A digital micrometre VOGEL DIN 862 with a resolution of 0.01 mm was used, and three points were marked in the middle of the panel of the specimen at the beginning of the test. TS was determined, following Equation (4), as an average of 10 samples per WHP board type.

(4) $$\mathrm{Thickness}\;\mathrm{Swelling}\;\left(\%\right)=\frac{E_{1}-E_{0}}{E_{0}}\times 100\%,$$

where *E*_0_ is the thickness at the beginning of the test, and *E*_1_ is the thickness after immersion in water for 24 h.

#### 2.2.7. Mechanical Testing of WHP Boards

A flexural test was performed by a three-point bending test, according to the European standard 12089 [47]. Three samples per WHP board type, of dimensions 150 × 75 × 15 mm, were tested. A universal mechanical test machine was used. The span of the supporting members was 10 mm. The load cell used was 5 kN and a cross-head speed of 10 mm/min was used. Displacement must be limited to 14.6 mm, as that was the maximum registrable value. Flexural stress was determined according to Equation (5):

(5) $$\mathrm{Flexural}\;\mathrm{stress}\;\left(N/{\mathrm{mm}}^{2}\right)=\frac{3\;FL}{2bd^{2}},$$

where *F* is the load (N), *L* is the span (mm), *b* is the width of the test piece (mm), and *d* is the thickness of the test piece (mm).

Modulus of Rupture (MOR) was calculated according to RILEM TFR1 [48] using Equation (5). In the case of pulp WHP board, the MOR value was calculated as the flexural stress sustained by the specimen at the maximum load. However, in the case of staple WHP, MOR was calculated as the flexural stress sustained by the specimen when the displacement value was 10% of the span supports (10 mm).

#### 2.2.8. Thermal Conductivity of WHP Boards

Finally, the thermal conductivity of the samples was analysed by a *PHYWE* high-insulating house, coupled with five thermocouples of type K (NiCr–Ni) [49]. It consisted of a direct comparison with an insulation of known thermal conductivity [50,51,52]. Samples were dried at 40 °C, for a minimum of 24 h, to achieve constant mass before testing them. Measurements were taken under steady-state conditions.

## 3. Results

### 3.1. WH Petiole Characterization

**Figure 3:**
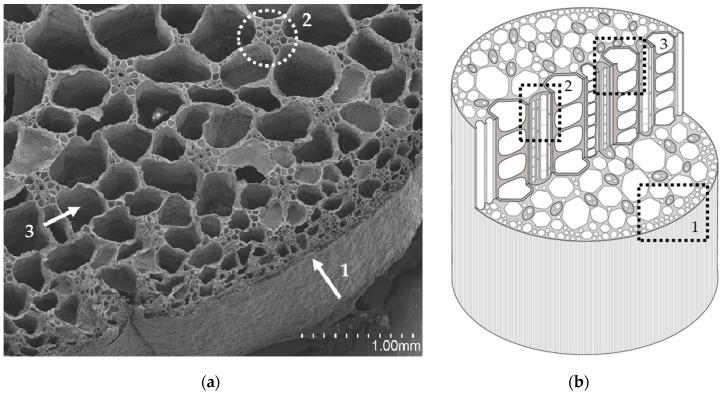
(**a**) General internal structure of the petiole section, SEM view: epidermal cells (1), “fibre unit” (2) and aerenchyma tissue (3); (**b**) General scheme of the petiole section: epidermal cells (1), “fibre unit” (2) and aerenchyma tissue (3).

**Figure 4:**
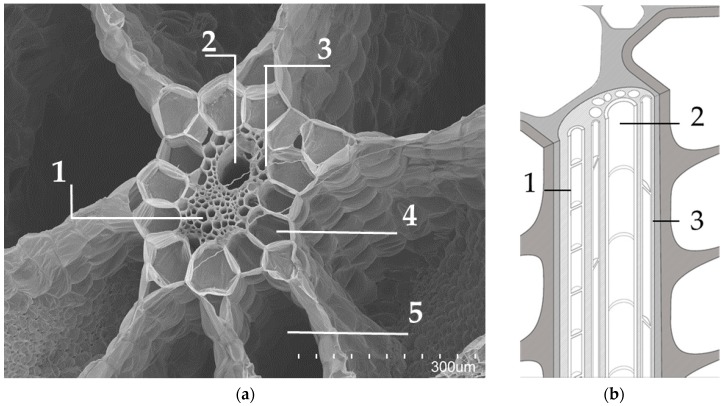
(**a**) Internal structure of the “fibre unit” of the petiole section, SEM view. “Fibre unit” composed of Vascular bundle (Phloem (1); Xylem (2); Companion cell (3)), surrounded by parenchymatous cells (4) and aerenchyma tissue (5); (**b**) Vascular bundle sketch (Phloem (1); Xylem (2); Companion cell (3)).

**Figure 5:**
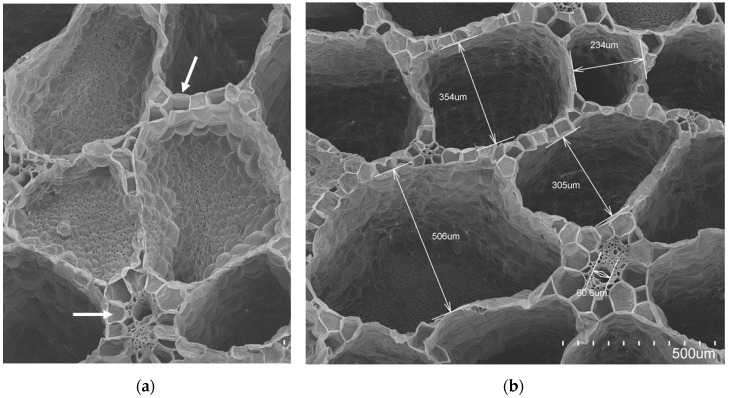
Internal structure of the petiole section, SEM view. Parenchymatous cells (**a**) and aerenchyma tissue (**b**), measured in four air chambers.

Figure 3, Figure 4, and Figure 5 display transversal sections of the petioles, which are circles measuring 10–15 mm in diameter in long leaves. The outer part is composed of a row of epidermal cells (Figure 1), and few external parenchyma cell layers.

**Figure 6:**
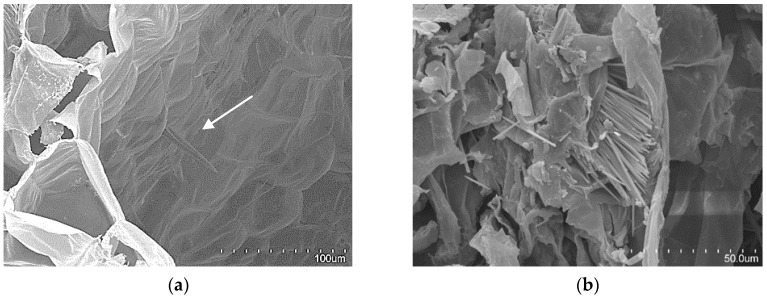
Aerenchyma tissue SEM image, showing sclereid cells projecting into air space (**a**). Needle-like crystals of oxalate calcium (**b**).

From a global view (Figure 1), a petiole section is constituted of 20–40 “fibre units” (about 320 µm) which can be manually separated. A detailed “fibre unit” is shown in Figure 4. Under the microscope, these “fibre units” are formed by vascular bundles, surrounded by scarce parenchyma and aerenchyma tissue. Vascular bundles are constituted of two parts: phloem (Figure 4.1) and xylem (Figure 4.2). Phloem is composed of sieve tubes and small companion cells. Xylem tissue consists of xylem vessels (empty and dried cells of about 60 µm in diameter, see Figure 4.2) and tracheids (empty and dried cells of about 20 µm in diameter, see Figure 4.3). Sclereid cells (Figure 6a) project out of tracheids. Besides these, there are living xylem parenchyma cells (Figure 4a and Figure 5a). Sclereids can be observed arising from aerenchyma cells and projecting into air spaces (Figure 6a). Aerenchyma (Figure 5b) is a special tissue of large, empty, and rather polygonal cells (filled with up to 300–550 µm of air). Finally, needle-like crystals of oxalate calcium (Figure 6b) are very frequent. 

Table 1 displays a summary of the physical characterization of both WHP particle sizes. In terms of moisture content, both types had the same values. On the contrary, differences in density and water absorption were found, in such a way that pulp WHP shows lower bulk density and higher water absorption than staple WHP.

In terms of chemical characterization (Table 2), WHP was mainly composed of holocellulose and hot water-soluble materials. Based on the chemical composition results (Table 2), no artificial matrix was used for the particleboard manufacture.

### 3.2. WHP-Panels Characterization

**Figure 7:**
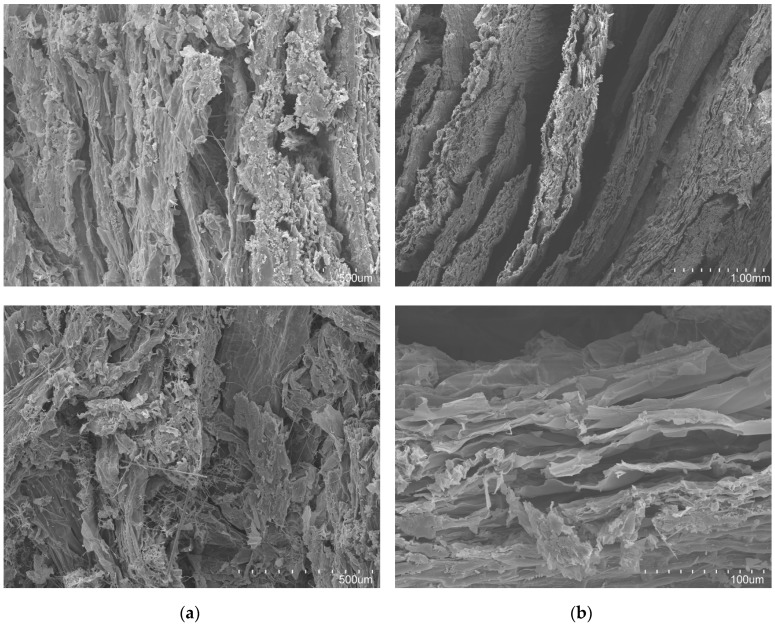
Internal structure of the WHP board, SEM view: pulp WHP panel (**a**) and staple WHP panel (**b**).

Table 3 shows a summary of the physical characteristics of the WHP boards, and Figure 7 displays the internal structure of the WHP-panels. Staple WHP had a lower density and moisture content than pulp WHP. However, pulp WHP showed a higher water absorption capacity. Despite this, its small particle size provoked a better stability dimension after water immersion than staple WHP. In the case of staple WHP, the high value dispersion of thickness swelling is related to the rugged surface of the material (Table 3). 

Pulp WHP panels presented a packing density structure (Figure 7a), and crushed parenchymatous tissues were shown, in addition to parts of aerenchyma tissues. Staple WHP (Figure 7b) had big pores, and so it had a higher open porosity. In addition, the whole WHP was shown and the aerenchyma tissues were complete and compressed, making the porosity volume higher. Many cellulose microfibrils with a diameter of 1 µm [53] were shown in the pulp WHP panels (Figure 7a), while there were no microfibrils in the case of the staple WHP panels (Figure 7b).

**Figure 8:**
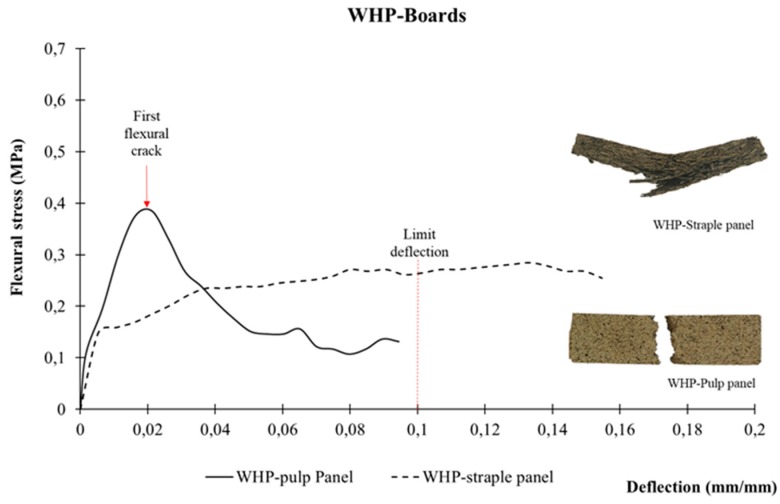
Flexural test: Stress–strain (mm/mm) curve of WH boards.

In both cases, the manufactured boards were self-supporting. Their flexural stress-deflection curves are shown in Figure 8. Staple WHP board had a ductile behaviour, reaching a higher flexural stress value in its plastic state. On the contrary, pulp WHP had a rigid performance and its flexural stress values fell after the first flexural stress crack. 

Table 3 shows the Modulus of Rupture (MOR) values, determined according to the RILEM standard [48]. In the case of staple WHPs, the boards had higher deflection values and were not even completely broken at the end of the test. The final appearance of the samples after the test is presented in Figure 8. Due to this, and in accordance with the standard [48], the deformations were limited to 10% of the span. Despite their rigid behaviour, pulp WHP panels had better mechanical properties. In fact, pulp WHP panels reached an MOR value 2.5 times higher than that of the staple WHP boards.

Finally, both WHP boards presented a good thermal insulation performance. In this sense, staple WHP had lower thermal conductivity than pulp WHP (Table 3).

## 4. Discussion

As was expected, the chemical composition of WHP favoured particle cohesion and the self-supporting properties of the panels. The WHP was mainly cellulose and hemicellulose (pentosans, xylenes, and polyholosides) which are polysaccharides with very good adhesive properties, used as industrial glues. They are water soluble, depending on the temperature and pH of the water [6]. In addition, heterogalactans—sulphated polysaccharides identical to seaweed thickeners—were identified in WHP [54], increasing the adhesive quality. 

While cellulose and hemicellulose are binding components, the lining is a polyalcohol, which, while not an adhesive macromolecule, confers other properties (i.e., rigidity) [55,56]. The WHP had lower lignin content, compared with previous results where the whole plant had been utilized [57]. Although the WH chemical composition varies, depending on the part of the plant [28,29,31,58,59], it is clear that WH is a low-lignin material, compared to other plants used for binder-less board manufacturing, such as kenaf, cotton stalks, or coconut, which have 9%, 26%, and 45% lignin, by weight, respectively [60,61,62]. For the same reason, unlike previous WH studies [33,34], the low levels of lignin in the petiole (compared with other plant fibres) [5,7] confirm that it is not necessary to perform de-lignification processes. Furthermore, the low lignin content justifies not requiring heat application in pre-treatments to obtain the “fibre unit” of the WH. Thus, the production of WHP boards is highly energy efficient, compared to other bio-based panels [60,61,62,63,64] 

The anatomical study of the petiole fibre confirmed that its optimal application is as a thermal insulation material. WH has the peculiarity of possessing one of the biggest aerenchyma known in the world [65]. WH aerenchyma are natural sets of empty cells with thin polysaccharide walls [66], with an organisation resembling bubble wrap (Figure 6). In previously studied WH panels [31,33,34], the internal pore structure of the WH was clogged by the matrices used. These matrices have a higher thermal conductivity than air and, therefore, had poor behaviour as a thermal insulator. Additionally, the mentioned authors applied higher compaction to their panels than in our case. The procedure, followed in the literature [31,33,34], destroyed the internal pore structure and eliminated the best potential characteristic of WH as a raw material. The aerenchymas are typical of aquatic plants but, in the case of WH, they are very abundant, forming its main biomass. Therefore, the WH structure of pores and air gaps is superior to that of other plant fibres studied as thermal insulation materials [5,7,67,68].

On the other hand, the parenchymatous cells and aerenchyma tissue are lighter than the “fibre unit” (see Figure 5 and Table 1), as they are composed of empty cells while the “fibre unit” is composed of lignin, which is a hard and strong material [69]. Therefore, two types of WHP material, with different chemical compositions, could be utilized. As a result, during the manual crushing process, aerenchyma tissues (Figure 5) are easily broken into smaller sizes (pulp) (Figure 1a) helped by the sclereid cells, comprising many needle-like crystals of calcium oxalate (Figure 6b); while larger sizes (staple) (Figure 1b) were integrated into the “fibre unit” and their surrounding aerenchymas (Figure 4). All of the above physical, mechanical, and thermal board properties were clearly affected by particle size.

In terms of bulk density, the staple WHP was lighter than pulp WHP, due to its poor packing density (as shown in Figure 7a). Compared with other bio-based thermal insulation materials [1,2,10], both boards had higher bulk-density values, due to the manufacture process. Commercial bio-based insulation materials are commonly manufactured as nonwoven material. This type of processing is not possible to achieve with WH, due to its poor lignin content. Contrarily, unlike the mentioned commercial insulation materials, the WH board was self-supporting. So, in fact, it was not necessary to follow a similar procedure to that of commercial materials.

As aforementioned, WHP pulp is comprised of the aerenchyma tissue cells, which are rich in cellulose, hemicellulose, and polyholosides, which are very hydrophilic macromolecules [53,65]. As a result, pulp WHP board had a higher water absorption capacity (1.25 times more) than staple WHP board. In the presence of water, the boards capture water and increase their volume individually by a chemical process [66,69]. For their part, the “fibre units” are comprised of microscopic tubes, such as the vessels of the xylem and phloem of the vascular bundles (Figure 4). Through these tubes, water penetrates by capillary action and remains there. In fact, the differences between capillarity and the chemical hydration process are also the reason for the thickness swelling results. As presented in Table 3, the staple WHP board had 1.75 times worse stability in dimension than pulp WHP panels. This is because, when the “fibre units” are full of water, their dimension increases. Contrarily, the pulp hydration process is a chemical process (namely, cellulose and hemicellulose hydration), and so the variability in dimension is lower. Dimensional variability must be considered, due to possible related pathologies in a building’s construction. On the other hand, water absorption capacity favours hydrothermal interior comfort. Additionally, this property could pose a problem in tropical contexts, where the relative humidity is high. Moisture content affects thermal-insulating performance where water thermal conductivity values are higher than air.

Finally, particle size also affects mechanical behaviour (Figure 8). Even though both binder-less WH panels were self-supporting, they had differing performances. Staple WHP had a ductile behaviour because the “fibre units” are dispersed randomly, and so it works like a nonwoven fibre panel [67,70,71]. However, pulp WHP panels had a rigid behaviour, similar to other pulp panels based on other plant fibres [42,70]. Notwithstanding, the Modulus of Rupture values of the pulp WHP panel were higher than that of the staple WHP, due to the external surface of petioles being mainly composed of lignin. As mentioned above, lignin does not have cohesive properties, and so it clearly influences the mechanical behaviour [69].

## 5. Conclusions

In the construction sector, an interest in bio-based insulation materials has been increasing, due to their contribution to sustainable development. Currently, a great deal of research into the use of biomass-like insulation material is being carried out. However, there has been only scarce investigation into Water Hyacinth (WH) biomass, even though it is a very aggressive and invasive plant. In fact, it has been increasing global awareness, due to its rapid growth.

To date, the use of WH as a building material has been inefficient, due to it being tested without a proper comprehension of the anatomy of its internal structure and chemical composition. In this paper, an analysis in depth of the internal structure and chemical make-up of WHP has allowed us to determine the potential applications of WHP in the construction sector. In addition, panels based on WH were tested to determine their physical, chemical, mechanical, and thermal properties. Based on the results obtained, it was concluded that:

1. The WHP low lignin content makes it possible to manufacture self-supporting binder-less WHP panels without requiring a heat energy procedure. The subsequent low energy involved in their manufacture makes them more sustainable, due to a reduced footprint.
2. Their peculiar structure of larger aerenchymas encapsulates air more efficiently, compared to other plant fibres. This is one reason why the WHP is an excellent raw material for thermal insulation production. Thermal conductivities of 0.047–0.065 W/mK were achieved by using pulp and staples, respectively.
3. The grinding process leads to particles of different size and microstructural composition. Pulp is mainly composed of aerenchyma tissue, while the staple is composed of “fibre units” and their surrounding aerenchymas. As a result, the physical, mechanical, and thermal properties of the boards are clearly affected by the particle size.
  - In this sense, staple WHP panels are 1.21 times lighter than pulp WHP panels.
  - In addition, pulp WHP panels have 2.5 times better Modulus of Rupture and 1.75 times better stability dimension behaviour than staple panels. Contrarily, staple WHP panels have 1.37 times better thermal insulation properties than pulp WHP panels, due to staple panels having a lower packing density, as was shown by SEM.

In summary, this research has shown that WHP has a competitive performance as an insulation material, due to its low lignin content and internal porous structure. Previously, the WH petiole was used much like other plant fibres in the building sector, without taking account its chemical and anatomical characterization. This paper demonstrates that the previous WH panels can be optimized, thanks to a comprehension of the chemical and internal structure of WHP. 

It has been demonstrated that detailed knowledge of the chemical composition and (anatomical) microstructure of water hyacinth “fibres”, obtained from the plant petioles, leads to an effective manufacture (low cost, low energy, binder-less—no artificial glue or matrix) of sustainable thermal insulation panels with reasonably good thermal conductivity.

## Figures and Tables

**Table 1:** Physical characterization of WH petiole (WHP).

WHP Size	Density (g/cm^3^)		Moisture Content (%)		Water Absorption (%)	
	Deviation		Deviation		Deviation	
Pulp	0.625	(±0.14)	7.82	(±1.87)	540.82	(±42.99)
Staple	0.834	(±0.17)	7.94	(±1.63)	441.83	(±64.74)

**Table 2:** Chemical characterization of Water Hyacinth (WH) petiole (WHP).

Sample	Holocellulose	Hot Water-Soluble	Pentosan	Lignin	Soluble in Neutral Organic Solvent
WHP	37.95 wt.%	36.11 wt.%	5.9 wt.%	5.84 wt.%	4.34 wt.%

**Table 3:** WHP boards characterization.

Sample	Density (g/cm^3^)	Moisture Content (%)	Water Absorption (%)	Thickness Swelling (%)	MOR (MPa)	Thermal Conductivity (W/mK)
pulp WHP	305.25 (±22.08)	8.63 (±0.79)	555.20 (±0.08)	57.68 (±10.34)	0.548 (±0.125)	0.065 (±0.00077)
staple WHP	251.23 (±12.35)	11.84 (±0.82)	450.51 (±0.08)	101.05 (±38.29)	0.215 (±0.095)	0.047 (±0.002474)

## References

[1] Asdrubali F., D’alessandro F., Schiavoni S. (2015). A review of unconventional sustainable building insulation materials. Sustain. Mater. Technol..

[2] Liu L., Li H., Lazzaretto A., Manente G., Tong C., Liu Q., Li N. (2017). The development history and prospects of biomass-based insulation materials for buildings. Renew. Sustain. Energy Rev..

[3] Palumbo M., Lacasta A.M., Holcroft N., Shea A., Walker P. (2016). Determination of hygrothermal parameters of experimental and commercial bio-based insulation materials. Constr. Build. Mater..

[4] Ali M. (2012). Natural fibres as construction materials. J. Civ. Eng. Constr. Technol..

[5] Faruk O., Bledzki A.K., Fink H.-P.P., Sain M. (2012). Biocomposites reinforced with natural fibres: 2000–2010. Prog. Polym. Sci..

[6] Gowthaman S., Nakashima K., Kawasaki S. (2018). A state-of-the-art review on soil reinforcement technology using natural plant fibre materials: Past findings, present trends and future directions. Materials.

[7] Ramesh M., Palanikumar K., Reddy K.H. (2017). Plant fibre based bio-composites: Sustainable and renewable green materials. Renew. Sustain. Energy Rev..

[8] Bordoloi S., Kashyap V., Garg A., Sreedeep S., Wei L., Andriyas S. (2018). Measurement of mechanical characteristics of fibre from a novel invasive weed: A comprehensive comparison with fibres from agricultural crops. Measurement.

[9] Bhuvaneshwari M., Sangeetha K. (2017). Effect of Blending Ratio of Water Hyacinth Fibres on the Properties of Needle Punched Nonwoven Fabrics. Int. J. Tech. Res. Appl..

[10] Schiavoni S., D’alessandro F., Bianchi F., Asdrubali F. (2016). Insulation materials for the building sector: A review and comparative analysis. Renew. Sustain. Energy Rev..

[11] Volf M., Diviš J., Havlík F. (2015). Thermal, moisture and biological behaviour of natural insulating materials. Energy Procedia.

[12] Liuzzi S., Sanarica S., Stefanizzi P. (2017). Use of agro-wastes in building materials in the Mediterranean area: A review. Energy Procedia.

[13] Pavelek M., Prajer M., Trgala K., Pavelek M., Prajer M., Trgala K. (2018). Static and Dynamic Thermal Characterization of Timber Frame/Wheat (Triticum Aestivum) Chaff Thermal Insulation Panel for Sustainable Building Construction. Sustainability.

[14] (2016). UNDP Senegal Technology transfer: Typha-based thermal insulation material production in senegal. UNDP Proj. Doc..

[15] Krus M., Theuerkorn W., Großkinsky T., Künzel H. (2014). New sustainable and insulating building material made of cattail. Nord. Symp. Build. Phys..

[16] Vejeliene J., Gailius A., Vejelis S., Vaitkus S., Balciunas G. Development of Thermal Insulation from Local Agricultural Waste. Proceedings of the VIII International Conference Environmental Engineering.

[17] Luamkanchanaphan T., Chotikaprakhan S., Jarusombati S. (2012). A Study of Physical, Mechanical and Thermal Properties for Thermal Insulation from Narrow-leaved Cattail Fibres. APCBEE Procedia.

[18] Téllez T.R., López E., Granado G., Pérez E., López R., Guzmán J. (2008). The Water Hyacinth, Eichhornia crassipes: An invasive plant in the Guadiana River Basin (Spain). Aquat. Invasions.

[19] Coetzee J.A., Hill M.P., Ruiz-Téllez T., Starfinger U., Brunel S. (2017). Monographs on invasive plants in Europe N° 2: Eichhornia crassipes (Mart.) Solms. Bot. Lett..

[20] Ali I., Jayaraman K., Bhattacharyya D. (2014). Implications of fibre characteristics and mat densification on permeability, compaction and properties of kenaf fibre panels. Ind. Crops Prod..

[21] (1981). Regional Research Laboratory of Commonwealth Regional (Asia/Pacific) Rural Technology Programme Possibilities of utilization of water hyacinth for making water hyacinth-cement boards. Report of the Second Review Meeting on Management of Water Hyacinth.

[22] Gosh S.R., Goswami T., Nombiar M.K.C., Chaliha B.P., Baruah J.N. Investigation on water hyacinth (Eichhornia crassipes) for making water hyacinth-cement boards. Proceedings of the International Conference on Water Hyacinth.

[23] Xu Y., Xu J., Yang H., Pu Q., Jiang L. (2013). Water Hyacinth Fibre Lightweight Concrete.

[24] Saleh H.M.M. (2014). Stability of cemented dried water hyacinth used for biosorption of radionuclides under various circumstances. J. Nucl. Mater..

[25] Na-Ayudhya B.I. (2016). Comparison of compressive and splitting tensile strength of autoclaved aerated concrete (AAC) containing water hyacinth and polypropylene fibre subjected to elevated temperatures. Mater. Struct..

[26] Marques M.L., Luzardo F.H.M., Velasco F.G., González L.N., Da Silva E.J., De Lima W.G. (2016). Compatibility of vegetable fibres with Portland cement and its relationship with the physical properties. Bras. Eng. Agríc. Ambient..

[27] Viwatsakpol S. (2014). Mortar Reinforced With Water Hyacinth Fibre.

[28] Methacanon P., Weerawatsophon U., Sumransin N., Prahsarn C., Bergado D.T. (2010). Properties and potential application of the selected natural fibres as limited life geotextiles. Carbohydr. Polym..

[29] Bordoloi S., Yamsani S.K., Garg A., Sreedeep S., Borah S. (2015). Study on the efficacy of harmful weed species Eicchornia crassipes for soil reinforcement. Ecol. Eng..

[30] Vardhan H., Bordoloi S., Garg A., Sreedeep S. (2017). Compressive strength analysis of soil reinforced with fibre extracted from water hyacinth. Eng. Comput..

[31] Saha M. (2011). Mechanical Characterization of Water Hyacinth Reinforced Polypropylene Composites.

[32] Tumolva T., Ortenero J., Kubouchi M. Characterization and Treatment of Water Hyacinth Fibres for NFRP Composites. Proceedings of the XIX International Conference of Composite Materials.

[33] Jaktorn C., Jiajitsawat S. (2014). Production of Thermal Insulator from Water Hyacinth Fibre and Natural Rubber Latex Energy Research & Promotion Center, Faculty of Sciences, Research Network & Innovation Development of Smart Materials for Energy, Sensors and Bio-resources. Int. J. Sci..

[34] Chimma T. (2001). The Feasibility Study of Cement Board Made by Mixing Water Hyacinth with Cement.

[35] Chatveera B., Nimityongskul P., Utyrt Y. (2013). Use of Water Hyacinth Fibre as Randomly-Oriented Reinforcement in Roofing Sheets. Eng. Appl. Sci. Res..

[36] Madurwar M.V., Ralegaonkar R.V., Mandavgane S.A. (2013). Application of agro-waste for sustainable construction materials: A review. Constr. Build. Mater..

[37] TAPPI 257 cm-02 (2012). Standard Test Method for Sampling and Preparing Wood for Analysis.

[38] TAPPI T 204 om-88 (2007). Standard Test Method for Solvent Extractives of Wood and Pulp.

[39] TAPPI T 207 cm-99 (1999). Standard Test Method for Water Solubility of Wood and Pulp.

[40] Wise L.E., Murphy M., D Adieco A.A. (1946). A chlorite holocellulose, its fractionation and bearing on summative wood analysis and studies on the hemicellulloses. Pap. Trade J..

[41] Sluiter A., Hames B., Ruiz R., Scarlata C., Sluiter J., Templeton D., Crocker D. (2008). Determination of Structural Carbohydrates and Lignin in Biomass: Laboratory Analytical Procedure (LAP) (Revised July 2011).

[42] Ardanuy M., Claramunt J., Toledo Filho R.D. (2015). Cellulosic fibre reinforced cement-based composites: A review of recent research. Constr. Build. Mater..

[43] EN 1602 (2013). European Standard Test Method for Thermal Insulating Products for Building Applications—Determination of the Apparent Density.

[44] EN 322 (1994). European Standard Test Method for Wood-Based Panels—Determination of Moisture Content.

[45] EN 12087 (2013). European Standard Test Method for Thermal Insulating Products for Building Applications—Determination of Long Term Water Absorption by Immersion.

[46] EN 317 (1994). European Standard Test Method for Particleboards and Fibreboards—Determination of Swelling in Thickness after Immersion in Water.

[47] EN 12089 (2013). European Standard Test Method for Thermal Insulating Products for Building Applications—Determination of Bending Behaviour.

[48] RILEM (1984). RILEM TC Test for the determination of modulus of rupture and limit of proportionality of thin fibre reinforced cement sections. RILEM Recommendations for the Testing and Use of Constructions Materials.

[49] PHYWE Series of Publications P 3.6.03-00 (2012). Insulation House. Laboratory Experiments.

[50] Herrero del Cura S. (2016). Influencia de la Dosificación y Granulometría del Caucho de Neumático Fuera de uso (NFU) y de las Domensiones Físicas en las Propiedades Térmicas, Acústicas y Mecánicas de Placas de Mortero de yeso y Caucho.

[51] Barbero-Barrera M.M., Flores-Medina N., Pérez-Villar V. (2017). Assessment of thermal performance of gypsum-based composites with revalorized graphite filler. Constr. Build. Mater..

[52] Navacerrada M.A., Fernandez P., Díaz C., Pedrero A. (2003). Thermal and Acoustic properties af aluminium foams manufactured by the infiltration process. Appl. Acoust..

[53] Sundari M.T., Ramesh A., Thiripura Sundari M., Ramesh A. (2011). Isolation and characterization of cellulose nanofibres from the aquatic weed water hyacinth-Eichhornia crassipes. Carbohydr. Polym..

[54] Dantas-Santos N., Gomes D.L., Costa L.S., Cordeiro S.L., Costa M.S.S.P., Trindade E.S., Franco C.R.C., Scortecci K.C., Leite E.L., Rocha H.A.O. (2012). Freshwater Plants Synthesize Sulfated Polysaccharides: Heterogalactans from Water Hyacinth (Eicchornia crassipes). Int. J. Mol. Sci..

[55] Bordoloi S., Hussain R., Garg A., Sreedeep S., Zhou W.-H. (2017). Infiltration characteristics of natural fibre reinforced soil. Transp. Geotech..

[56] Bordoloi S., Garg A., Sreedeep S. (2016). Potential of Uncultivated, Harmful and Abundant Weed as a Natural Geo-Reinforcement Material. Adv. Civ. Eng. Mater..

[57] Abdel-Fattah A.F., Abdel-Naby M.A. (2012). Pretreatment and enzymic saccharification of water hyacinth cellulose. Carbohydr. Polym..

[58] Hasan M.R., Chakrabarti R. (2009). Floating aquatic macrophytes—Water hyacinths. Use of Algae and Aquatic Macrophytes as Feed in Small-Scale Aquaculture—A Review.

[59] Zhou W., Zhu D., Langdon A., Li L., Liao S., Tan L. (2009). The structure characterization of cellulose xanthogenate derived from the straw of Eichhornia crassipes. Bioresour. Technol..

[60] Panyakaew S., Fotios S. (2011). New thermal insulation boards made from coconut husk and bagasse. Energy Build..

[61] Zhou X., Zheng F., Li H., Lu C. (2010). An environment-friendly thermal insulation material from cotton stalk fibres. Energy Build..

[62] Xu J., Sugawara R., Widyorini R., Han G., Kawai S. (2004). Manufacture and properties of low-density binderless particleboard from kenaf core. J. Wood Sci..

[63] Pickering K.L., Efendy M.G.A., Le T.M. (2016). A review of recent developments in natural fibre composites and their mechanical performance. Compos. Part A Appl. Sci. Manuf..

[64] Boukhattem L., Boumhaout M., Hamdi H., Benhamou B., Nouh F.A. (2017). Moisture content influence on the thermal conductivity of insulating building materials made from date palm fibres mesh. Constr. Build. Mater..

[65] Bhattacharya A., Haldar S., Chatterjee P.K. (2015). Geographical distribution and physiology of water hyacinth (Eichhornia crassipses)—the invasive hydrophyte and a biomass for producing xylitol. Int. J. ChemTech Res..

[66] Keegstra K. (2010). Plant cell walls. Plant Physiol..

[67] Yan L., Kasal B., Huang L. (2016). A review of recent research on the use of cellulosic fibres, their fibre fabric reinforced cementitious, geo-polymer and polymer composites in civil engineering. Compos. Part B Eng..

[68] Gurunathan T., Mohanty S., Nayak S.K. (2015). A review of the recent developments in biocomposites based on natural fibres and their application perspectives. Compos. Part A Appl. Sci. Manuf..

[69] Chen H. (2014). Chemical Composition and Structure of Natural Lignocellulose. Biotechnology of Lignocellulose.

[70] Claramunt J., Ventura H., Fernández-Carrasco L., Ardanuy M. (2017). Tensile and Flexural Properties of Cement Composites Reinforced with Flax Nonwoven Fabrics. Materials.

[71] Benfratello S., Capitano C., Peri G., Rizzo G., Scaccianoce G., Sorrentino G. (2013). Thermal and structural properties of a hemp-lime biocomposite. Constr. Build. Mater..

